# Supplementary material for: Sex- and Gamete-Specific Patterns of X Chromosome Segregation in a Trioecious Nematode
Source: Curr Biol. 2018 Jan 8;28(1):93–99.e3. doi: 10.1016/j.cub.2017.11.037 (PMC5772170; doi:10.1016/j.cub.2017.11.037)
Supplement: Document S1. Figures S1–S3 [file mmc1.pdf]

Current Biology, Volume *28*

Supplemental Information

Sex- and Gamete-Specific Patterns of X Chromosome Segregation in a Trioecious Nematode

Sophie Tandonnet, Maureen C. Farrell, Georgios D. Koutsovoulos, Mark L. Blaxter, Manish Parihar, Penny L. Sadler, Diane C. Shakes, and Andre Pires-daSilva

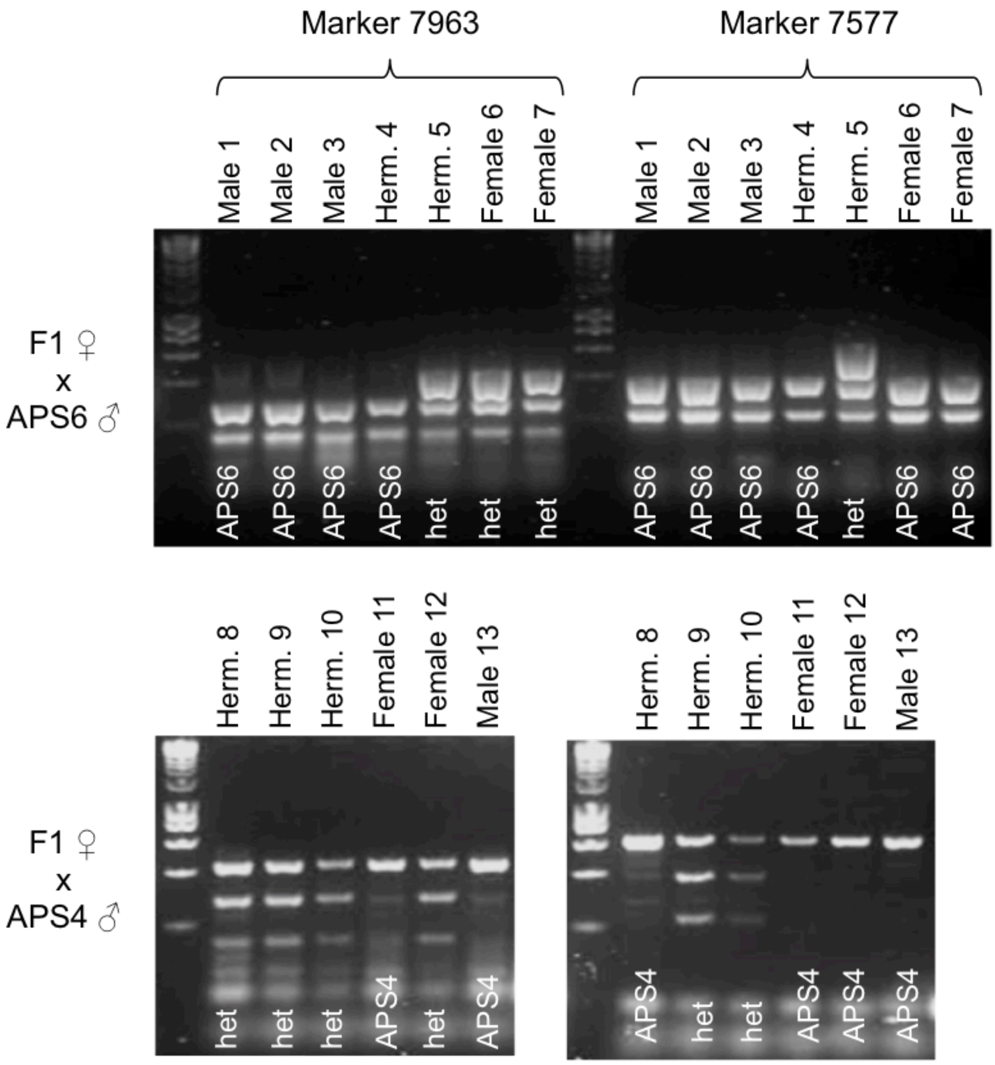

Figure S1, related to Figure 3B and C. Example of X chromosome genotyping of F2s generated from crosses between F1 hybrid $X_{APS4}X_{APS6}$ females and either APS6 (upper panel) or APS4 (lower panel) males.

From this genotyping, we can infer that some crossovers have occurred during female oogenesis as some F2 XX progeny do not display the same genotype across all the markers genotyped. The gel depicts only the two rightmost X markers (see figure 3A) as the crossovers were frequently observed between these markers (probably due to the subtelomeric position of the marker 7577). Genotypes are reported under the gel pictures. 'het' stands for heterozygous. Numbers indicate individual animals.

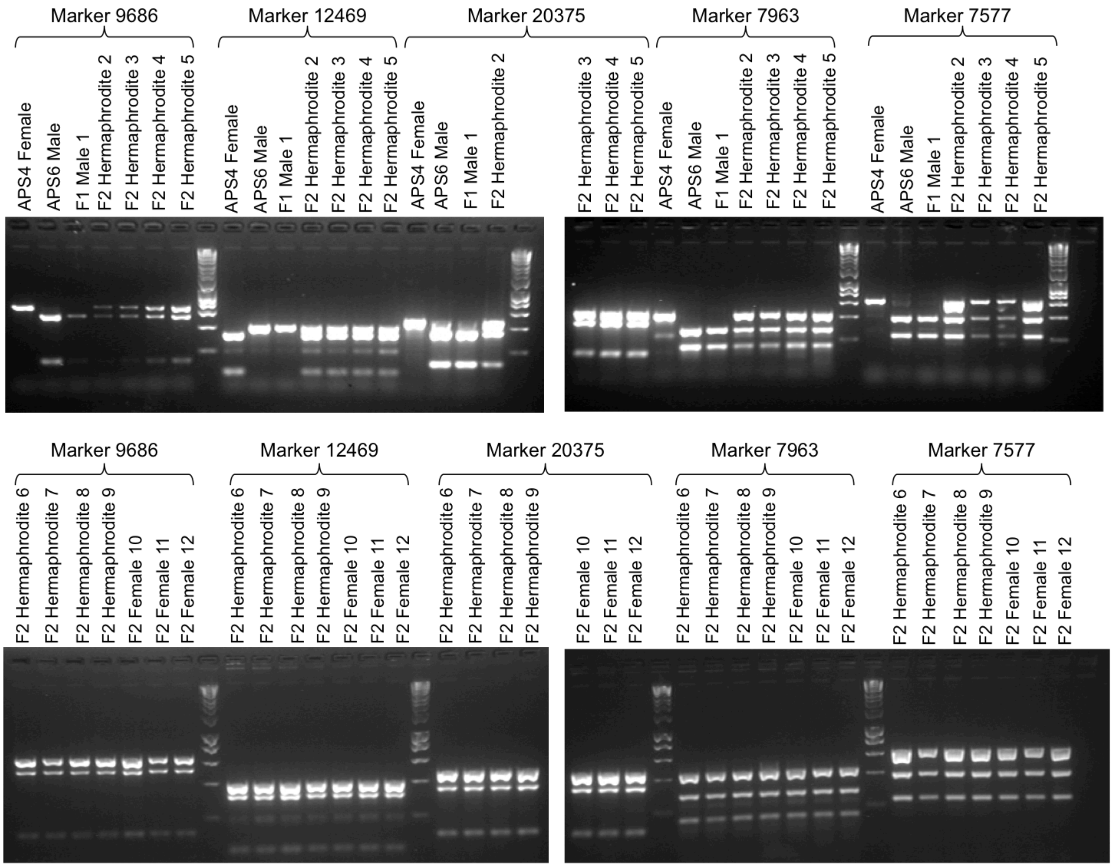

Figure S2, related to Figure 3B and D. Example of X chromosome genotyping using amplification, digestion and electrophoresis of parental individuals and F2 females and hermaphrodites produced by selfing F1 hybrid hermaphrodites.

F2 XX progeny (females and hermaphrodites) produced by hybrid F1 hermaphrodites are systematically heterozygous across the 5 X markers genotyped. Numbers indicate individual animals.

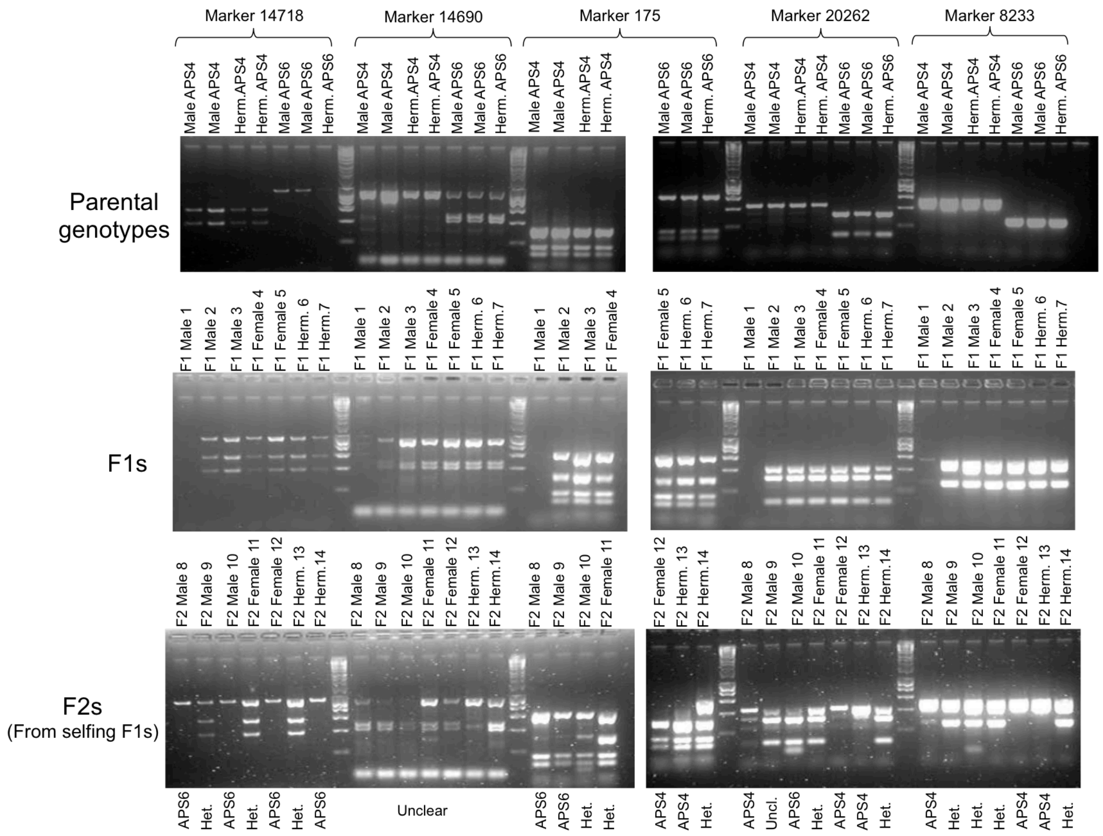

Figure S3, related to Figure 3. Example of LG4 (autosome) genotyping using amplification, digestion and electrophoresis.

F2 genotypes are reported under the gel pictures. ‘Het.’ and ‘Uncl.’ stand for heterozygous and unclear genotypes, respectively. Numbers indicate individual animals.
