# Supplementary material for: Sex- and Gamete-Specific Patterns of X Chromosome Segregation in a Trioecious Nematode
Source: Curr Biol. 2018 Jan 8;28(1):93–99.e3. doi: 10.1016/j.cub.2017.11.037 (PMC5772170; doi:10.1016/j.cub.2017.11.037)
Supplement: Document S2. Article plus Supplemental Information [file mmc4.pdf]

Report

Current Biology

Sex- and Gamete-Specific Patterns of X Chromosome Segregation in a Trioecious Nematode

Graphical Abstract

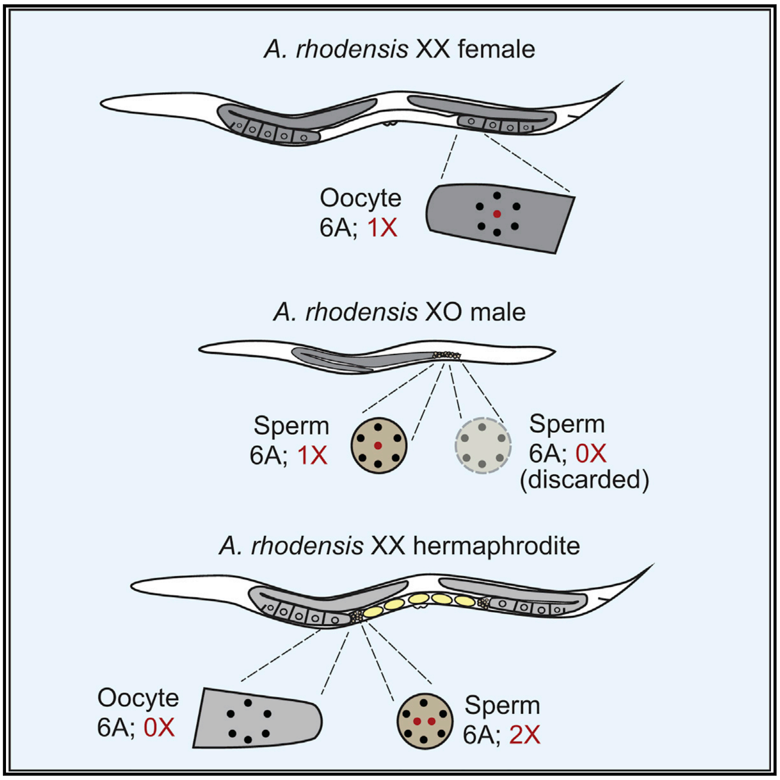

Highlights

- Crosses between *A. rhodensis* hermaphrodites and males generate only male progeny
- Hermaphrodites generate mostly nullo-X oocytes and diplo-X sperm
- Following normal Mendelian genetics, XX females produce haplo-X oocytes
- In cross-progeny, sons always inherit the X chromosome from the father

Authors

Sophie Tandonnet, Maureen C. Farrell, Georgios D. Koutsovoulos, ..., Penny L. Sadler, Diane C. Shakes, Andre Pires-daSilva

Correspondence

andre.pires@warwick.ac.uk

In Brief

Tandonnet et al. show that the X chromosomes segregate differently between sexes and type of gametogenesis in the three-sexed nematode *A. rhodensis*: hermaphrodites produce diplo-X sperm and nullo-X oocytes, whereas females produce haplo-X oocytes. Furthermore, it has a unique form of X chromosome heritability, from father to son.

Tandonnet et al., 2018, Current Biology *28*, 93–99
January 8, 2018 © 2017 The Author(s). Published by Elsevier Ltd.
https://doi.org/10.1016/j.cub.2017.11.037

Current Biology
Report

CellPress

Sex- and Gamete-Specific Patterns of X Chromosome Segregation in a Trioecious Nematode

Sophie Tandonnet,[1] Maureen C. Farrell,[2] Georgios D. Koutsovoulos,[3,4] Mark L. Blaxter,[3] Manish Parihar,[1,5] Penny L. Sadler,[2] Diane C. Shakes,[2] and Andre Pires-daSilva[1,6,*]
[1]School of Life Sciences, University of Warwick, Coventry CV4 7AL, UK
[2]Department of Biology, College of William and Mary, Williamsburg, VA 23187, USA
[3]Institute of Evolutionary Biology, University of Edinburgh, Edinburgh EH9 3JT, UK
[4]Present address: INRA, UMR 1355 Institute Sophia Agrobiotech, 06903 Sophia Antipolis, France
[5]Present address: Department of Molecular Medicine and Institute of Biotechnology, University of Texas Health Science Center at San Antonio, San Antonio, TX 78229, USA
[6]Lead Contact
*Correspondence: andre.pires@warwick.ac.uk
https://doi.org/10.1016/j.cub.2017.11.037

SUMMARY

Three key steps in meiosis allow diploid organisms to produce haploid gametes: (1) homologous chromosomes (homologs) pair and undergo crossovers; (2) homologs segregate to opposite poles; and (3) sister chromatids segregate to opposite poles. The XX/XO sex determination system found in many nematodes [1] facilitates the study of meiosis because variation is easily recognized [2–4]. Here we show that meiotic segregation of X chromosomes in the trioecious nematode *Auanema rhodensis* [5] varies according to sex (hermaphrodite, female, or male) and type of gametogenesis (oogenesis or spermatogenesis). In this species, XO males exclusively produce X-bearing sperm [6, 7]. The unpaired X precociously separates into sister chromatids, which co-segregate with the autosome set to generate a functional haplo-X sperm. The other set of autosomes is discarded into a residual body. Here we explore the X chromosome behavior in female and hermaphrodite meioses. Whereas X chromosomes segregate following the canonical pattern during XX female oogenesis to yield haplo-X oocytes, during XX hermaphrodite oogenesis they segregate to the first polar body to yield nullo-X oocytes. Thus, crosses between XX hermaphrodites and males yield exclusively male progeny. During hermaphrodite spermatogenesis, the sister chromatids of the X chromosomes separate during meiosis I, and homologous X chromatids segregate to the functional sperm to create diplo-X sperm. Given these intra-species, intra-individual, and intra-gametogenesis variations in the meiotic program, *A. rhodensis* is an ideal model for studying the plasticity of meiosis and how it can be modulated.

RESULTS AND DISCUSSION

Genetic Crosses Suggest Unorthodox Patterns of Meiotic X Chromosome Segregation that Are Both Sex and Gamete Specific

Genetic crosses and cytological analyses show that *Auanema rhodensis* XO males produce exclusively haplo-X sperm [6, 7]. Crosses between males and females yield almost only XX progeny (hermaphrodites or females) [8], which implies that most female oocytes carry a single X (Figure 1A). However, without morphological genetic markers, it had been impossible to distinguish between self- and outcross progeny in crosses between males and hermaphrodites. Using our new, morphologically marked strain containing a recessive dumpy mutation, we performed crosses between dumpy hermaphrodites and wild-type males. The resulting cross-progeny were easily distinguished by their non-dumpy phenotype. Contrary to our expectations, all cross-progeny were male (306 normal non-dumpy males scored from 10 hermaphrodite/male crosses).

Because male sperm have a single X, this result implies that XX hermaphrodites produce oocytes without an X (nullo-X oocytes) (Figure 1A). Furthermore, because self-fertilizing hermaphrodites produce 90%–95% XX self-progeny [8–10], their nullo-X oocytes must be fertilized by hermaphrodite sperm that are predominantly diplo-X (Figure 1A).

Cytological Analysis of Meiotic X Chromosome Segregation

During Hermaphrodite Oogenesis, Both X Chromosomes Appear to Segregate to the First Polar Body

Our crossing results predicted specific cytological consequences. We hypothesized that during oogenesis in *A. rhodensis* hermaphrodites, unorthodox segregation patterns of the X chromosome would result not only in anaphase figures with unequal amounts of chromatin but also in non-standard numbers of DAPI-stained bodies aligned at the metaphase plate due to potential alterations in X chromosome pairing. We examined meiotically dividing oocytes labeled with a combination of DAPI-staining and anti-tubulin antibodies (see STAR Methods) and compared the patterns in *A. rhodensis* females

This is an open access article under the CC BY license (http://creativecommons.org/licenses/by/4.0/).

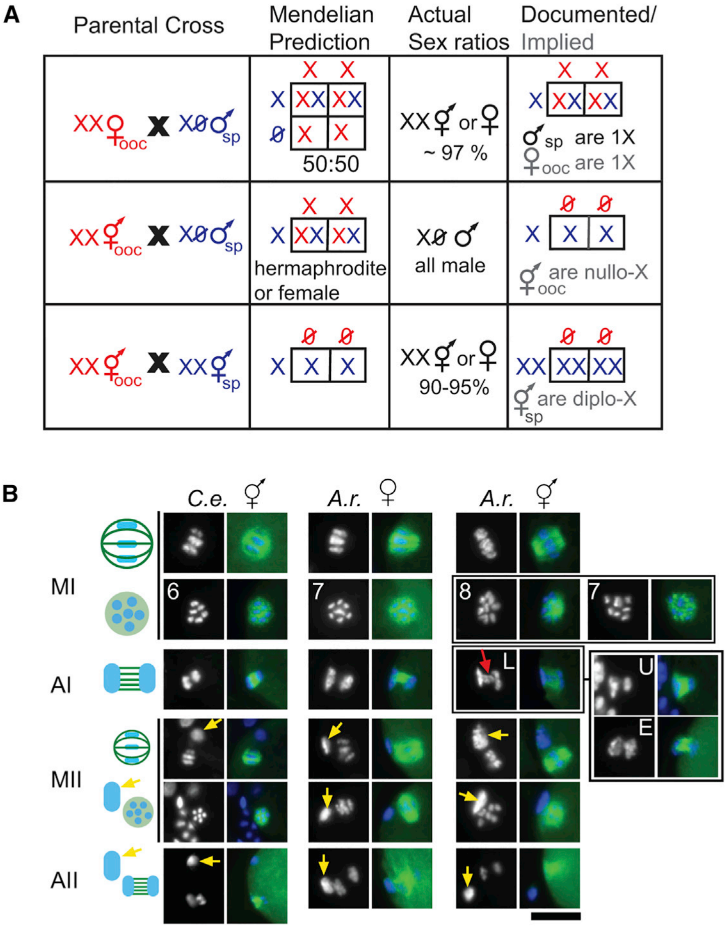

Figure 1. Patterns of Chromosome Segregation during *A. rhodensis* Oocyte Meiosis

(A) Crosses between XX females and XO males (upper row) generate mostly XX progeny, because males mainly produce haplo-X sperm. This result implies that female oocytes are haplo-X. Crosses between XX hermaphrodites and XO males (middle row) result only in male progeny, implying the production of nullo-X oocytes by hermaphrodites. Self-fertilization of an XX hermaphrodite (lower row) results mostly in XX progeny, implying that sperm are diplo-X. In red are the gametes produced during oogenesis (ooc) and in blue gametes produced during spermatogenesis (sp).

(B) Chromosome segregation patterns were imaged in fixed, meiotic one-cell embryos. Chromosomes were stained with DAPI (blue), and microtubules were labeled with the anti-tubulin antibody (green). Schematics of the meiotic divisions are shown in the left column. Metaphase spindles are shown in two orientations; either from the side (upper) or viewed down the pole to show the metaphase plate (lower). For *A. rhodensis* (*A.r.*) hermaphrodites, metaphase I plates with 8 and 7 DAPI-staining bodies are shown as well as anaphase I figures with lagging chromosomes (L), unequal chromosome segregation (U), and a rare example of an equal chromosome segregation (E). The red arrow shows lagging chromosomes during anaphase I in *A. rhodensis* hermaphrodites. The yellow arrows show polar bodies. *C.e.*, *C. elegans*; MI, metaphase I; AI, anaphase I; MII, metaphase II; AII, anaphase II. Scale bar, 10 μm.

(C) Results of blindly scored anaphase figures during oogenesis.

C

Species and sex	Stage of oocyte meiosis	Lagging chromosome	Equal segregation	Unequal segregation
C. elegans hermaphrodite	Anaphase I (n=15)	0%	100%	0%
	Anaphase II (n=6)	0%	100%	0%
A. rhodensis female	Anaphase I (n=11)	9%	91%	0%
	Anaphase II (n=3)	0%	100%	0%
A. rhodensis hermaphrodite	Anaphase I (n=36)	31%	5%	64%
	Anaphase II (n=29)	0%	100%	0%

and hermaphrodites to the well-established patterns in *C. elegans* [11–14].

During *C. elegans* oogenesis, chromosome condensation occurs over an extended period during late meiotic prophase [11]. Thus, it is relatively easy to observe metaphase I figures with six bivalents (five autosomes and one X). In contrast, chromosome condensation in *A. rhodensis* occurs rapidly between the end of meiotic prophase and metaphase I (data not shown), and thus scorable metaphase I figures with well-resolved chromosomes were relatively rare. When we did observe them (3/3), the metaphase I figures in *A. rhodensis* females had seven DAPI-stained structures, consistent with genomic analyses that suggest *A. rhodensis* has six autosomes and an X (S.T., unpublished data). In the oocytes of *A. rhodensis* females, chromosome segregation patterns during both anaphase I and anaphase II appear equal, as similar size and intensity of DAPI signals were observed (Figure 1B), although we did find examples of lagging chromosomes during early anaphase I (Figure 1C). In contrast, analyses of hermaphrodite oocytes in *A. rhodensis* revealed two key differences. First, the metaphase I figures were scored as having either seven (4/17) or eight (13/17) DAPI-stained structures, although it was unclear whether some of the "7s" could have been "8s." Observing eight structures is consistent with the presence of X chromosomes that have failed to pair or recombine. Second, anaphase I figures typically exhibited either lagging chromosomes or unequal chromosome segregation (Figures 1B and 1C). Consistent with the unequal pattern of chromosome segregation, the first polar bodies were disproportionally large. In contrast, anaphase II

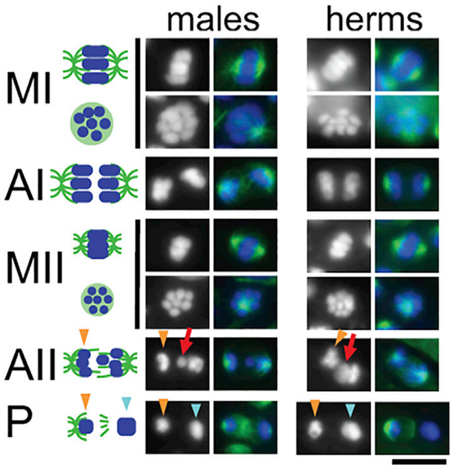

Figure 2. Patterns of Chromosome Segregation during *A. rhodensis* Spermatocyte Meiosis

Chromosome segregation patterns were imaged in isolated and fixed male and hermaphrodite gonads. Chromosomes were stained with DAPI (blue), and microtubules were labeled with anti-tubulin antibody (green). A schematic of the meiotic divisions is shown in the left column. Metaphase spindles are shown in two orientations to either show the spindle or viewed down the pole to show the metaphase plate. The red arrows indicate lagging chromosomes during anaphase II. The orange arrowheads indicate the chromatin mass of the future residual body during anaphase II and the partitioning (P) phase. The light blue arrowheads indicate the larger chromatin mass of the future sperm. Meiotic stage abbreviations are as in Figure 1B. Scale bar, 5 μm.

figures were always equal. Taken together, the frequent observation of an additional DAPI-staining body in metaphase I plates of *A. rhodensis* hermaphrodite oocytes and the unequal divisions observed during anaphase I suggest a model in which the X chromosomes of hermaphrodite oocytes fail to pair and/or recombine during meiotic prophase and then are partitioned to the first polar body during anaphase I.

During Hermaphrodite Spermatogenesis, X Chromatids Appear to Separate Precociously in Meiosis I and Then Differentially Partition to the Functional Sperm

Previously, we showed that sperm production in *A. rhodensis* hermaphrodites differs from that in *C. elegans*, because *A. rhodensis* hermaphrodites produce sperm from discrete clusters of spermatogonial cells—both simultaneously and continuously along with oocytes [15]. In addition, *A. rhodensis* hermaphrodites, like *A. rhodensis* males, produce only two rather than four functional sperm during meiosis [6, 7]. We had previously assumed that hermaphrodite sperm, like those in *A. rhodensis* males, contained a single X [7]. However, if *A. rhodensis* hermaphrodites routinely produce nullo-X oocytes, the production of predominantly XX progeny by self-fertilizing hermaphrodites predicts that XX hermaphrodites are making diplo-X rather than haplo-X sperm. To test this prediction, we examined meiotically dividing spermatocytes in *A. rhodensis* hermaphrodites and compared them with patterns that we previously described in males [6, 7].

In *A. rhodensis* XO male spermatocytes, the X chromatids separate precociously during meiosis I, resulting in each secondary spermatocyte receiving a single X chromatid [6]. During anaphase II, the lagging X chromatid invariably ends up in the functional male sperm, whereas the other chromosomal complement is discarded in a “residual body” (Figure 2) [6, 7]. In XX *A. rhodensis* hermaphrodites, clusters of synchronously dividing spermatocytes arise from discrete clusters of spermatogonial cells [15]. Analysis of 520 hermaphrodite gonads yielded 16 clusters with anaphase II stage spermatocytes. Within each cluster, all scorable (oriented parallel to the slide and whose tubulin patterns could be distinguished from the tubulin of the underlying oocyte) spermatocytes (1–9 per cluster; 74 total) exhibited a lagging, potentially unresolved, DAPI-staining chromatin mass that was roughly twice the size of those in anaphase II male spermatocytes (Figure 2). In the same set of specimens, we identified 17 clusters with post-meiotic, partitioning stage sperm and, in each scorable pair (1–6 per cluster; 44 total), the functional sperm appeared to have more DNA than the tubulin-containing residual body (Figure 2). These observations, taken together, provide cytological evidence that the hermaphroditic sperm most likely contain two X chromosomes.

Genotyping of X Chromosome SNP Markers Reveals Patterns of Chromosome Segregation in Female Oocyte Meiosis and Hermaphrodite Spermatocyte Meiosis

Based on the sex ratios observed in the crosses, we inferred that the unpaired or lagging chromosomes observed in the cytological studies were X chromosomes. However, DAPI staining alone does not directly test whether these are X chromosomes or whether they are undergoing meiotic recombination. To address these questions, we tracked the segregation patterns of X chromosomes using single-nucleotide polymorphisms (SNPs) between two strains of *A. rhodensis* (APS4 and APS6). For this analysis, we selected 5 polymorphic markers distributed along the length of the X chromosome (Figure 3A; STAR Methods) and used them to genotype the X chromosome in filial generation 2 (F2) individuals produced either by crossing hybrid ($X_{APS4}X_{APS6}$) females with males from the original inbred strains or by selfing hybrid ($X_{APS4}X_{APS6}$) hermaphrodites.

Female Oocyte Meiosis

Intra-specific hybrid ($X_{APS4}X_{APS6}$) F1 females were crossed with males of one of the parental strains (e.g., X_{APS6}). Genotypic analysis of the resulting F2 XX progeny yielded the expected 1:1 ratio of homozygous ($X_{APS6}X_{APS6}$) to heterozygous ($X_{APS6}X_{APS4}$) markers in the X chromosome (chi-square 3.37, df 1, p value = 0.07; Figure 3C; Data S1; STAR Methods). We also identified 12 crossovers where, in a single individual, some X chromosome markers were heterozygous and others homozygous (Figures 3B and 3C; Figure S1; Data S1). These data suggest conventional meiotic pairing and segregation of the X chromosome in *A. rhodensis* females.

Hermaphrodite Spermatocyte Meiosis

Following Mendelian segregation patterns, the X genotyping of F2 XX progeny produced by selfing hybrid F1 ($X_{APS4}X_{APS6}$) hermaphrodites would predict a 1:2:1 ratio of $X_{APS4}X_{APS4}$:$X_{APS4}X_{APS6}$:$X_{APS6}X_{APS6}$ progeny in XX F2s. However, all 82 F2 XX progeny genotyped were fully heterozygous (i.e., $X_{APS4}X_{APS6}$) for the five X chromosome markers (Figures 3B and 3D; Data S1; Figure S2). The complete lack of homozygosity for any markers implies that (1) no recombination between the X chromosomes took place during hermaphrodite spermatogenesis, and (2) the two X chromosomes in the diplo-X sperm are homologs, not

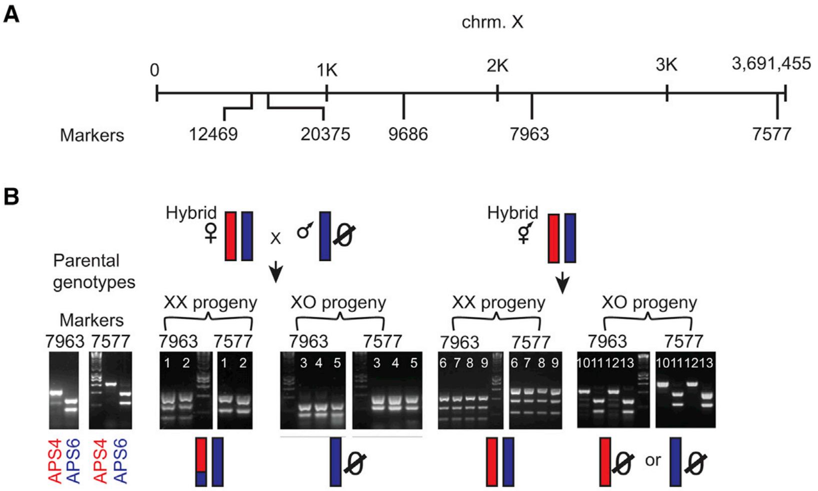

C Oocyte meiosis in hybrid females

	Progeny	N genotyped	Heterozygous	Homozygous	Crossovers
$X_{APS4}X_{APS6}$ female cross with X_{APS4} or X_{APS6} male	Female	13	26 (54%)	22 (46%)	6
	Hermaphrodite	22	50 (60%)	33 (40%)	6
	Total	35	76 (58%)	55 (42%)	12

D Spermatocyte meiosis in hybrid hermaphrodites

	Progeny	N genotyped	Heterozygous	Homozygous	Crossovers
$X_{APS4}X_{APS6}$ selfing hermaphrodite	Female	40	178 (100%)	0	0
	Hermaphrodite	42	196 (100%)	0	0
	Total	82	374 (100%)	0	0

E F1 male genotypes

	APS4 ♀ X APS6 ♂ cross		APS6 ♀ X APS4 ♂ cross	
F1 male genotype	APS4	APS6	APS4	APS6
Marker 12469	0	23	18	0
Marker 20375	0	19	20	0
Marker 9686	0	21	18	0
Marker 7963	0	20	15	0
Marker 7577	0	18	19	0

Figure 3. X Chromosome Markers and Genotyping Results

(A) Schematic view of the markers used to genotype the X chromosome.

(B) Left: genotyping profile of parental strains. Center: a hybrid female crossing with an APS6 male generates XX progeny with both homozygous and heterozygous X markers. Crossovers could be detected when the X of one individual was part heterozygous, part homozygous, as represented here by individuals 1 and 2. Male offspring resulting from the cross always inherited the X from their father. Right: X genotyping of individuals produced by hybrid selfing hermaphrodites reveals that the X chromosome remains heterozygous in XX individuals and hemizygous for each parental strain in males. Numbers in each gel lane represent individual animals. See also Figures S1–S3 for X and LG4 genotyping profiles.

(C) Genotype counts of F2 XX progeny from hybrid F1 crossed females. See also Figure S1.

(D) Genotype counts of F2 XX progeny from hybrid F1 selfing hermaphrodites. See also Figure S2.

(E) X chromosome genotyping of F1 males resulting from crosses between the APS4 and APS6 parental strains.

sisters. This X chromosome behavior is consistent with a model in which both X chromosomes of a hermaphrodite spermatocyte separate into sister chromatids in meiosis I and then both X chromatids segregate to the functional sperm in meiosis II (Figure 4D).

Importantly, this behavior was specific to the X chromosome, as genotyping of the autosome LG4, also across 5 markers (Data S1, sheet 2; Figure S3; STAR Methods), yielded a mix of homozygous and heterozygous markers (24 homozygous and 12 heterozygous markers). In addition, autosomal crossovers could be observed, as the genotype was not uniform across all markers for the same individual (Data S1, sheet 2).

Taken together, our combined cytological and genetic data indicate that the patterns of X chromosome segregation in XX animals differ both between female and hermaphrodite oogenesis and between oocyte meiosis and spermatocyte meiosis within hermaphrodites. Inferred patterns of segregation are depicted schematically in Figure 4. In female oocytes, X chromosomes pair, recombine, and segregate at metaphase I (MI), following a conventional meiosis scheme (Figure 4A). In hermaphrodite oocytes, both X chromosomes preferentially segregate to the polar body at metaphase I (Figure 4C). This is most readily explained by the failure in pairing and/or crossing over during prophase, which would lead to univalent X chromosomes at metaphase I. As observed in *C. elegans* [14], the resulting X univalents would be preferentially placed in the first polar body and thus eliminated. In the case of hermaphrodite spermatogenesis (Figure 4D), the co-segregation of two non-sister chromatids to the sperm indicates that the X chromosomes (1) fail to pair and/or undergo crossing over during prophase, and (2) the resulting X univalents undergo equational segregation at metaphase I (premature sister chromatid separation), as observed for male spermatocyte meiosis (Figure 4B). Because genetically identical X chromosomes segregated differentially between sexes and gametogenesis types, control of the meiosis modulations observed in *A. rhodensis* cannot lie in the X chromosome sequence per se. This implies that (1) the regulation of X chromosome pairing and/or crossovers must differ between the female and hermaphrodite oogenesis programs, and (2) a difference in the regulation of cohesion loss must occur in hermaphrodite spermatogenesis to explain the premature sister chromatid separation of the X.

Father-to-Son X Chromosome Inheritance

The predominant X segregation patterns of female and hermaphrodite meiosis depicted in Figure 4 do not provide a ready explanation regarding how rare XO males arise in cross-progeny

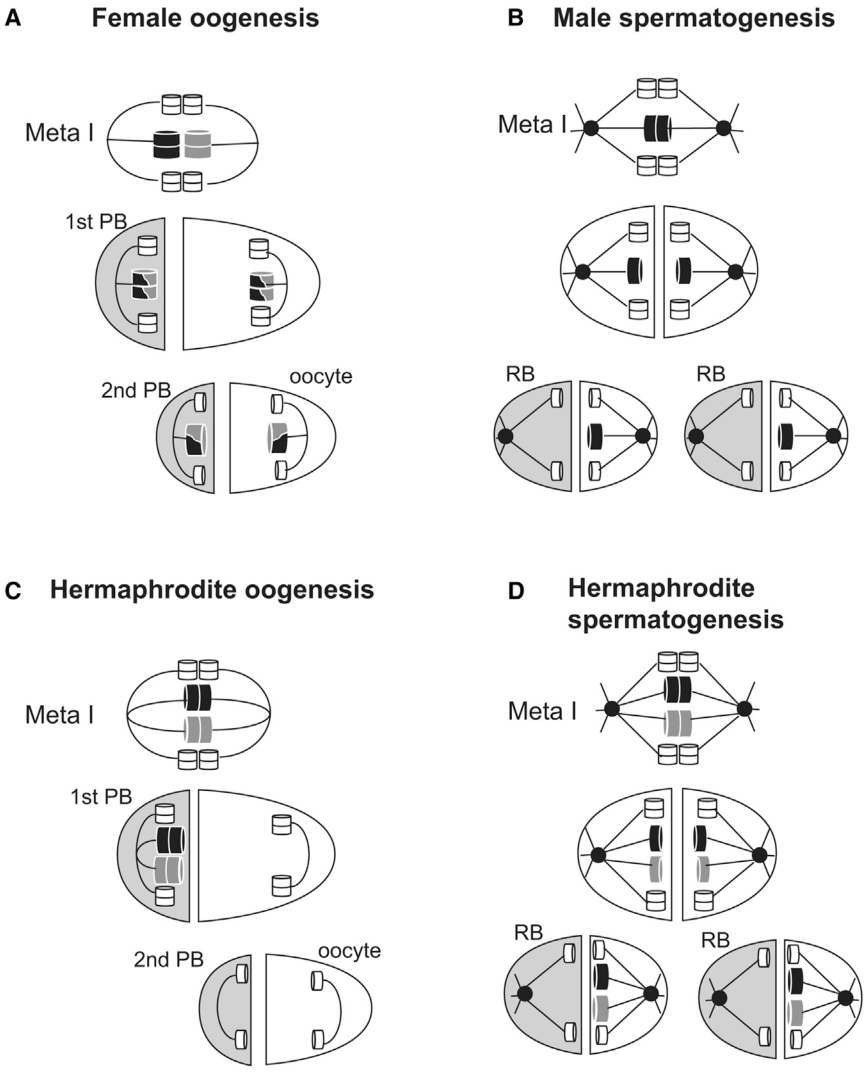

Figure 4. Simplified Model of the X Chromosome Segregation Mechanism in *A. rhodensis*

For a Figure360 author presentation of Figure 4, see the figure legend at https://doi.org/10.1016/j.cub.2017.11.037. Figure360▷

(A) In females, autosome (white cylinders) and X chromosome (darker and larger cylinders) dynamics follow the canonical segregation pattern, with pairing and crossover. Shaded cells are polar bodies (PBs). Lines represent microtubules.

(B) In XO males, the homologous autosomes segregate to different daughter cells in meiosis I, and sister chromatids separate in meiosis II. For the unpaired X chromosome, however, sister chromatids separate in meiosis I. In meiosis II, the X chromatids co-segregate with one autosome set to the functional sperm, whereas the other set of autosomes is discarded into a residual body (RB; shaded in gray). Black circles represent centrioles.

(C) Hermaphrodite oogenesis generates functional nullo-X oocytes. During meiosis I, the homologous X chromosomes are unpaired at the metaphase plate and, during anaphase I, all X chromatids segregate to the first polar body.

(D) Hermaphrodite spermatogenesis generates diplo-X sperm. During meiosis I, the homologous X chromosomes are unpaired at the metaphase plate and separate into sister chromatids. During meiosis II, both X chromatids (non-sisters) segregate to the functional sperm.

of XX females or in self-progeny of XX hermaphrodites. Thus, we genotyped the X chromosome of rare males produced by female/male crosses or by selfing hermaphrodites.

Males Produced by Male/Female Crosses

Sons resulting from female/male crosses always inherited the X markers of their father (~40 males genotyped across five X chromosome markers; Figure 3E; Data S1). As far as we know, this is the only example of a complete X chromosome transmission through the male lineage in a sexually reproducing context. This finding also implies that, during female meiosis, unusual meiotic divisions must sometimes generate nullo-X oocytes, presumably in a manner mechanistically similar to the routine production of nullo-X oocytes in hermaphrodites.

The atypical male-to-male transmission of the X chromosome in *A. rhodensis* is reminiscent of androgenesis, a type of reproduction that occurs in a conifer, a few ants and stick insects, and clams of the genus *Corbicula* (reviewed by [16]): the male inherits the genome solely from his father. As a consequence, this may lead to the genetic divergence of the female and male lineages over time [17]. However, in *A. rhodensis*, the father-to-son genetic inheritance is limited to the X chromosome, which is transmitted to all sexual morphs and has a chance to recombine in females, thus preventing the genetic divergence of the X between XO males and XX individuals.

One evolutionary consequence of this observation is that any beneficial mutations on the X will spread quickly through the population, as male carriers will transmit it to all their offspring, including their sons, which will, in turn, systematically pass it on. Additionally, as there is no crossover between the X chromosomes during hermaphrodite meiosis, this means that the *A. rhodensis* X chromosome has a very different recombinational and evolutionary trajectory from the *C. elegans* X. If X-linked genes control traits subject to selection, the maintenance of diversity in X chromosomes in XX nematode offspring of hermaphrodites could impact the colonizing ability of a single hermaphrodite nematode.

Males Produced by Selfing Hermaphrodites

Males produced by selfing $X_{APS4}X_{APS6}$ hermaphrodites either carried X_{APS4} or X_{APS6} (Figure 3B; Data S1). No crossovers were observed (100 genotypes from 21 males genotyped; Figure 3B; Data S1) and it is, therefore, possible that no recombination between the X homologs occurred. To explain the occurrence of male offspring from selfing hermaphrodites, we postulate that hermaphrodite spermatocytes sometimes divide to generate haplo-X rather than diplo-X sperm.

Intriguingly, selfing hermaphrodites regularly produce more males early in their broods [8], suggesting that the choice of the division pattern is developmentally regulated. Furthermore, because sperm within the hermaphrodite germline are produced in spermatogonial clusters [15], it may be that different clusters produce sperm with different X chromosome complements.

These observations indicate that the meiosis program is actively modulated within the same type of gametogenesis, generating a flexible system where the proportion of male offspring can be adjusted through regulation of the X chromosome segregation in both female and hermaphrodite mothers. The factors controlling this regulation, and thus the XO:XX sex ratio, could be environmental, and may reflect adaptation to the colonization ecology of *A. rhodensis*.

Concluding Remarks

The recent findings and data collected on *A. rhodensis* open the door to investigating the peculiarities and implications of its sex determination system, understanding mechanistically the processes that control X chromosome segregation, and exploring the evolutionary and population genetic consequences of the curious pattern of X chromosome inheritance. *A. rhodensis* is mutable, and screening for genetic loci that specifically affect female, hermaphrodite, or male X chromosome segregation (i.e., the proportion of male offspring generated) is feasible given the genetic and genomic resources we have generated. Particularly, *A. rhodensis* is an ideal model for studying the regulation of the meiotic process and how it can be altered within the same genetic context. We note that developmental context (hermaphrodite versus female) plays an important role in the modulation of meiotic processes affecting the X. For instance, XX animals that develop through a dauer larva stage always become hermaphrodites [18], whereas larvae that bypass this stage become females. What triggers this differential development and how it links with the meiotic process are still open questions.

STAR★METHODS

Detailed methods are provided in the online version of this paper and include the following:

- KEY RESOURCES TABLE
- CONTACT FOR REAGENT AND RESOURCE SHARING
- EXPERIMENTAL MODEL AND SUBJECT DETAILS
  - Nematode Strains and growth condition
- METHOD DETAILS
  - Genotyping of chromosomes
  - Crosses between hermaphrodites and males
  - Immunocytology
- QUANTIFICATION AND STATISTICAL ANALYSIS
  - Genotyping experiments
- DATA AND SOFTWARE AVAILABILITY

SUPPLEMENTAL INFORMATION

Supplemental Information includes three figures and one data file and can be found with this article online at https://doi.org/10.1016/j.cub.2017.11.037.

ACKNOWLEDGMENTS

G.D.K. was supported by a BBSRC PhD studentship and S.T. by a PhD training grant from CAPES/CNPq (201116/2014-6). A.P.-d.S. was supported by grants from the National Science Foundation (IOS 1122095) and BBSRC (BB/L019884/1) and University of Warwick start-up funds. D.C.S. was supported by a grant from the National Science Foundation (IOS 1122101). We thank Dr. Sally Adams, Dr. Pedro Robles Naharro, and Dr. James Stratford for many insightful conversations.

AUTHOR CONTRIBUTIONS

S.T., D.C.S., and A.P.-d.S. designed the study. M.C.F., P.L.S., and D.C.S. conducted the cell biology experiments. S.T., G.D.K., M.L.B., and M.P. generated the genetic markers. S.T. performed the genetic experiments. S.T., M.L.B., D.C.S., and A.P.-d.S. wrote the paper.

Received: October 24, 2017
Revised: November 13, 2017
Accepted: November 16, 2017
Published: December 21, 2017

REFERENCES

1. Pires-daSilva, A. (2007). Evolution of the control of sexual identity in nematodes. Semin. Cell Dev. Biol. *18*, 362–370.
2. Hodgkin, J., Horvitz, H.R., and Brenner, S. (1979). Nondisjunction mutants of the nematode *Caenorhabditis elegans*. Genetics *91*, 67–94.
3. Meneely, P.M., Farago, A.F., and Kauffman, T.M. (2002). Crossover distribution and high interference for both the X chromosome and an autosome during oogenesis and spermatogenesis in *Caenorhabditis elegans*. Genetics *162*, 1169–1177.
4. Rog, O., and Dernburg, A.F. (2013). Chromosome pairing and synapsis during *Caenorhabditis elegans* meiosis. Curr. Opin. Cell Biol. *25*, 349–356.
5. Kanzaki, N., Kiontke, K., Tanaka, R., Hirooka, Y., Schwarz, A., Müller-Reichert, T., Chaudhuri, J., and Pires-daSilva, A. (2017). Description of two three-gendered nematode species in the new genus *Auanema* (Rhabditina) that are models for reproductive mode evolution. Sci. Rep. *7*, 11135.
6. Shakes, D.C., Neva, B.J., Huynh, H., Chaudhuri, J., and Pires-daSilva, A. (2011). Asymmetric spermatocyte division as a mechanism for controlling sex ratios. Nat. Commun. *2*, 157.
7. Winter, E.S., Schwarz, A., Fabig, G., Feldman, J.L., Pires-daSilva, A., Müller-Reichert, T., Sadler, P.L., and Shakes, D.C. (2017). Cytoskeletal variations in an asymmetric cell division support diversity in nematode sperm size and sex ratios. Development *144*, 3253–3263.
8. Chaudhuri, J., Bose, N., Tandonnet, S., Adams, S., Zuco, G., Kache, V., Parihar, M., von Reuss, S.H., Schroeder, F.C., and Pires-daSilva, A. (2015). Mating dynamics in a nematode with three sexes and its evolutionary implications. Sci. Rep. *5*, 17676.
9. Félix, M.A. (2004). Alternative morphs and plasticity of vulval development in a rhabditid nematode species. Dev. Genes Evol. *214*, 55–63.
10. Farrell, M.C. (2015). An investigation of hermaphroditism in R. sp. SB347. MS thesis (College of William and Mary).
11. Albertson, D.G., and Thomson, J.N. (1993). Segregation of holocentric chromosomes at meiosis in the nematode, *Caenorhabditis elegans*. Chromosome Res. *1*, 15–26.
12. Golden, A., Sadler, P.L., Wallenfang, M.R., Schumacher, J.M., Hamill, D.R., Bates, G., Bowerman, B., Seydoux, G., and Shakes, D.C. (2000). Metaphase to anaphase (*mat*) transition-defective mutants in *Caenorhabditis elegans*. J. Cell Biol. *151*, 1469–1482.
13. Dumont, J., Oegema, K., and Desai, A. (2010). A kinetochore-independent mechanism drives anaphase chromosome separation during acentrosomal meiosis. Nat. Cell Biol. *12*, 894–901.

14. Cortes, D.B., McNally, K.L., Mains, P.E., and McNally, F.J. (2015). The asymmetry of female meiosis reduces the frequency of inheritance of unpaired chromosomes. eLife *4*, e06056.
15. McCaig, C.M., Lin, X., Farrell, M., Rehain-Bell, K., and Shakes, D.C. (2017). Germ cell cysts and simultaneous sperm and oocyte production in a hermaphroditic nematode. Dev. Biol. *430*, 362–373.
16. Schwander, T., and Oldroyd, B.P. (2016). Androgenesis: where males hijack eggs to clone themselves. Philos. Trans. R. Soc. Lond. B Biol. Sci. *371*, 20150534.
17. Fournier, D., Estoup, A., Orivel, J., Foucaud, J., Jourdan, H., Le Breton, J., and Keller, L. (2005). Clonal reproduction by males and females in the little fire ant. Nature *435*, 1230–1234.
18. Chaudhuri, J., Kache, V., and Pires-daSilva, A. (2011). Regulation of sexual plasticity in a nematode that produces males, females, and hermaphrodites. Curr. Biol. *21*, 1548–1551.
19. Stiernagle, T. (2006). Maintenance of *C. elegans*. WormBook, 1–11.
20. Church, D.L., Guan, K.L., and Lambie, E.J. (1995). Three genes of the MAP kinase cascade, *mek-2*, *mpk-1/sur-1* and *let-60 ras*, are required for meiotic cell cycle progression in *Caenorhabditis elegans*. Development *121*, 2525–2535.
21. Brenner, S. (1974). The genetics of *Caenorhabditis elegans*. Genetics *77*, 71–94.
22. Boyd, L., Guo, S., Levitan, D., Stinchcomb, D.T., and Kemphues, K.J. (1996). PAR-2 is asymmetrically distributed and promotes association of P granules and PAR-1 with the cortex in *C. elegans* embryos. Development *122*, 3075–3084.
23. Shakes, D.C., Wu, J.C., Sadler, P.L., Laprade, K., Moore, L.L., Noritake, A., and Chu, D.S. (2009). Spermatogenesis-specific features of the meiotic program in *Caenorhabditis elegans*. PLoS Genet. *5*, e1000611.

STAR★METHODS

KEY RESOURCES TABLE

REAGENT or RESOURCE	SOURCE	IDENTIFIER
Antibodies		
FITC-conjugated anti-α-tubulin DM1A	Sigma-Aldrich	F2168-.2ML
Bacterial and Virus Strains		
Escherichia coli OP50-1	Caenorhabditis Genetics Center	OP50-1
Chemicals, Peptides, and Recombinant Proteins		
NdeI	Promega	R6801
RsaI	Promega	R6371
ScaI	Promega	R6211
HaeIII	Promega	R6171
EcoRV	Promega	R6351
HinfI	Promega	R6201
GoTaq Green Master Mix	Promega	M7822
Proteinase K	Fisher Scientific	26160
PCR buffer (10X)	Sigma-Aldrich	P2317-5ML
Magnesium sulfate (NGM)	Sigma-Aldrich	208094
Cholesterol (NGM)	Sigma-Aldrich	C8503
Calcium Chloride (NGM)	Fisher Scientific	10171800
Potassium Dihydrogen Orthophosphate (NGM)	Fisher Scientific	10783611
Di-Potassium Hydrogen Orthophosphate Anhydrous (NGM)	Fisher Scientific	10375760
Sodium Chloride (NGM)	Fisher Scientific	10428420
Bacto Peptone (NGM)	BD	211677
Agar (NGM)	BD	214530
Nystatin	Fisher Scientific	10034587
Streptomycin sulfate	Melford Biolaboratories	S0148
Tris.HCl	Fisher Scientific	BP153-500
Tris.OH	Fisher Scientific	BP152-1
Bactotryptone	Fisher Scientific	BP1421-500
Cholesterol	Spectrum Chemical	CH135
Sodium Chloride	Fisher Scientific	BP358-212
Agar	Fisher Scientific	BP1423-500
DAPI	Sigma	D-9564
Potassium Chloride	EM Science	PX1405-1
Sodium Phosphate Na2HPO4	Fisher Scientific	S374-1
Magnesium Chloride	Fisher Scientific	BP9741
Calcium Chloride	Mallinckrodt	7722
Dextrose	DIFCO	0155-17-4
Deposited Data		
Complete results of X chromosome genotyping and autosomal genotyping	This paper	Data S1
Genetic markers	This paper; Mendeley Data	https://doi.org/10.17632/63d7rrrx28.3
Experimental Models: Organisms/Strains		
A. rhodensis, strain APS4	[18]	N/A
A. rhodensis, strains APS6 and APS19 (dumpy phenotype)	This paper	N/A
C. elegans: strain N2	Caenorhabditis Genetics Center, https://cbs.umn.edu/cgc/home	N2

(*Continued on next page*)

Continued		
REAGENT or RESOURCE	SOURCE	IDENTIFIER
Oligonucleotides		
X marker 9686: Forward: 5′-TGTCCTGACCCGCGTGTTGA-3′, Reverse: 5′-AACTGAGTTTGCAGCCCTGT-3′	IDT (custom DNA oligos)	N/A
X marker 12469: Forward: 5′-TGCAAGGCAGACGTCCCTTG-3′, Reverse: 5′-CCAATTCTTCGCTTATTGCCCG-3′	IDT (custom DNA oligos)	N/A
X marker 20375: Forward: 5′-ACCCTGCTGATCCTCGACTCG-3′, Reverse: 5′-AGGAGTCCCCAAACACCCCA-3′	IDT (custom DNA oligos)	N/A
X marker 7963: Forward: 5′-TGGTGGGGCTTGGAGTTCGA-3′, Reverse: 5′-ACGGCTGATGTTGACGCTCC-3′	IDT (custom DNA oligos)	N/A
X marker 7577: Forward: 5′-GTTGCACAAGCCCACACTGG-3′, Reverse: 5′-CGACCTTTCTCTTCCAGACATTGC-3′	IDT (custom DNA oligos)	N/A
Autosomal (LG4) marker 14718: Forward: 5′-CCGAAGCCACTT GGTGCTGT-3′, Reverse: 5′-CGTTCGAGCTGGGCGTGTAA-3′	IDT (custom DNA oligos)	N/A
Autosomal (LG4) marker 14690: Forward: 5′-CTGCAGCTCGTT TTGGCCGT-3′, Reverse: 5′-GGCACATAAGGGGGAGGCCA-3′	IDT (custom DNA oligos)	N/A
Autosomal (LG4) marker 175: Forward: 5′-GCTTCGTCAGCGC ACTGTCT-3′, Reverse: 5′-GTCGGCTGTTGCTTCTTCGGT-3′	IDT (custom DNA oligos)	N/A
Autosomal (LG4) marker 20262: Forward: 5′-GGTTTCGAGATTACC CGACGACG-3′, Reverse: 5′-CCAGCTGTCTTAAGATCCTACAGG-3′	IDT (custom DNA oligos)	N/A
Autosomal (LG4) marker 8233: Forward: 5′-TGCCGTAAAACCTG CATCCCC-3′, Reverse: 5′-TCGAGCCAACTCTTCCTCCTGT-3′	IDT (custom DNA oligos)	N/A

CONTACT FOR REAGENT AND RESOURCE SHARING

Further information and requests for resources and reagents should be directed to and will be fulfilled by the Lead Contact, Andre Pires-daSilva (andre.pires@warwick.ac.uk).

EXPERIMENTAL MODEL AND SUBJECT DETAILS

Nematode Strains and growth condition

We used two isolates of *Auanema rhodensis*, originally derived from a deer tick (strain SB347, Rhode Island, USA) [9] and from a dead tiger beetle (strain TMG33, West Virginia, USA; found in May 2012, GPS 38.230011, −81.762252) (T. Grana, personal communication). Inbred strains were generated by picking single hermaphrodite animals from populations derived from a self-fertilizing parent. The strain SB347, which underwent 50 rounds of bottlenecking of inbreeding, was subsequently renamed APS4. The strain TMG33, inbred for 11 rounds of bottlenecking, was renamed APS6. Strains were maintained at 20°C according to standard conditions as for *C. elegans* [19], either on MYOB agar (2.0 g/L NaCl, 0.55 g/L Tris.HCl, 0.24 g/L Tris.OH, 4.6 g/L Bactotryptone, 8 mg/L Cholesterol, 20 g/L Agar) [20] for cytological studies or Nematode Growth Medium (3 g/L Sodium chloride, 2.5 g/L bacto peptone, 17 g/L agar, 1 mM Magnesium Sulfate, 5 mg/L Cholesterol, 1 mM Calcium Chloride, 25 mM Potassium phosphate) [21] for molecular studies. Plates were seeded with the *Escherichia coli* streptomycin resistant strain OP50-1. For molecular studies, microbial contamination was prevented by adding 50 μg/mL of streptomycin and 10 μg/mL of nystatin to the Nematode Growth Medium (NGM).

METHOD DETAILS

Genotyping of chromosomes

To genotype the X chromosome and autosomal linkage group 4 (LG4), we used 5 polymorphic markers (SNPs) for each chromosome (Data S1). We generated these markers from a draft genome sequence for *A. rhodensis*, a genetic linkage map (S.T., unpublished data) and strain-specific sequences (RAD-seq markers). The markers were selected for the presence of a restriction enzyme site characteristic of one strain but not the other. Amplifications of the polymorphic regions were performed by single-worm PCRs followed by digestion of the products (see Key Resources Table and Data S1). Genomic DNA template was extracted by worm lysis by freezing (minimum 5 min) a single worm in 10 μL of 1X PCR buffer (see Key Resources Table) and, after thawing, adding 0.5 μL of proteinase K (20 mg/mL). Samples were incubated at 65°C for 60 min to lyse the worms and release the genomic DNA followed by enzyme inactivation at 95°C for 15min. The DNA samples were kept at −80°C for a minimum of 12 h before using. Each PCR reaction was performed in a total volume of 20 μL, using 2 μL of DNA, the GoTaq Green MasterMix (Promega) and 5 μM of each primer (see Key Resources Table). The following cycling conditions were applied: 95°C for 7 min, followed by 30-35 cycles of 15 s at 94°C, 30 s at 55°C, and 1 min at 72°C. The digestion of the PCR products was performed at 35°C for one to two hours. The genotype of each

marker was visualized by gel electrophoresis of the digested products. The markers were confirmed to be X-linked by genotyping intra-species hybrid F1 males (XO). As expected from hemizygosity in XO animals, F1 males always showed a single genotype for markers on the X chromosome.

Crosses between hermaphrodites and males

To distinguish hermaphrodite self-progeny from cross-progeny, we used morphologically-marked hermaphrodites (dumpy phenotype, strain APS19, caused by a recessive mutation). Ten crosses between a marked hermaphrodite and a wild-type APS4 male were performed. The offspring were scored according to their phenotype (dumpy versus wild-type) and gender at the adult stage. The female and hermaphroditic morphs were not distinguished.

Immunocytology

To obtain *A. rhodensis* adults of specific sexes, *A. rhodensis* hermaphrodites were isolated by selecting dauer larvae [9]. Males and females were isolated from early broods of *A. rhodensis* hermaphrodites [8] and the gonads of females were secondarily verified by the absence of spermatogonia [15].

To isolate meiotically dividing spermatocytes and meiotic one-cell embryos for analysis, hermaphrodites, males, mated females were dissected in Edgar's buffer [22] on ColorFrost Plus slides (Fisher Scientific) coated with poly-L-lysine (Sigma-Aldrich). Samples were freeze-cracked in liquid nitrogen and fixed in -20°C methanol. Anti-tubulin labeling was done as previously described [23] using 1:100 (0.025 mg/mL) FITC-conjugated anti-α-tubulin DM1A (Sigma-Aldrich). Slides were mounted with Fluoro-Gel II (Electron Microscopy Sciences) containing 6-diamidino-2-phenylindole (DAPI) and visualized under epi-illumination using an Olympus BX60 microscope.

QUANTIFICATION AND STATISTICAL ANALYSIS

Genotyping experiments

43 F1 males, 14 females and 20 hermaphrodites produced by either APS4 female/APS6 male crosses (denoted "forward cross" in Data S1) or by APS6 female/APS4 male crosses ("Reciprocal cross") were genotyped across the 5 X-linked markers, following the genotyping methodology explained above. The same procedure was used to genotype 24 F2 males, 13 F2 females and 23 F2 hermaphrodites produced by hybrid F1 females crossed with either APS4 or APS6 males were genotyped (denoted as backcrosses in Data S1). Likewise, 21 F2 males, 40 F2 females and 42 F2 hermaphrodites produced by F1 selfing hybrid hermaphrodites resulting from either APS4 female/APS6 male crosses ("Forward cross") or from APS6 female/APS4 male crosses ("Reciprocal cross") were genotyped.

Autosomal genotyping of LG4 was performed on 11 F1 individuals (5 males, 3 females, 3 hermaphrodites) resulting from either an APS4 female / APS6 males cross or its reciprocal and 10 F2s produced by selfing hybrid F1 hermaphrodites.

Data S1 contains all the information on the individuals and markers genotyped. Failed and ambiguous genotyping is indicated by red and yellow cells.

DATA AND SOFTWARE AVAILABILITY

Draft genome sequences and the genetic map have not yet been published and are not yet on public databases. The genetic markers derived from them are available as Mendeley Data (https://doi.org/10.17632/63d7rrrx28.3).

Current Biology, Volume *28*

Supplemental Information

Sex- and Gamete-Specific Patterns of X Chromosome Segregation in a Trioecious Nematode

Sophie Tandonnet, Maureen C. Farrell, Georgios D. Koutsovoulos, Mark L. Blaxter, Manish Parihar, Penny L. Sadler, Diane C. Shakes, and Andre Pires-daSilva

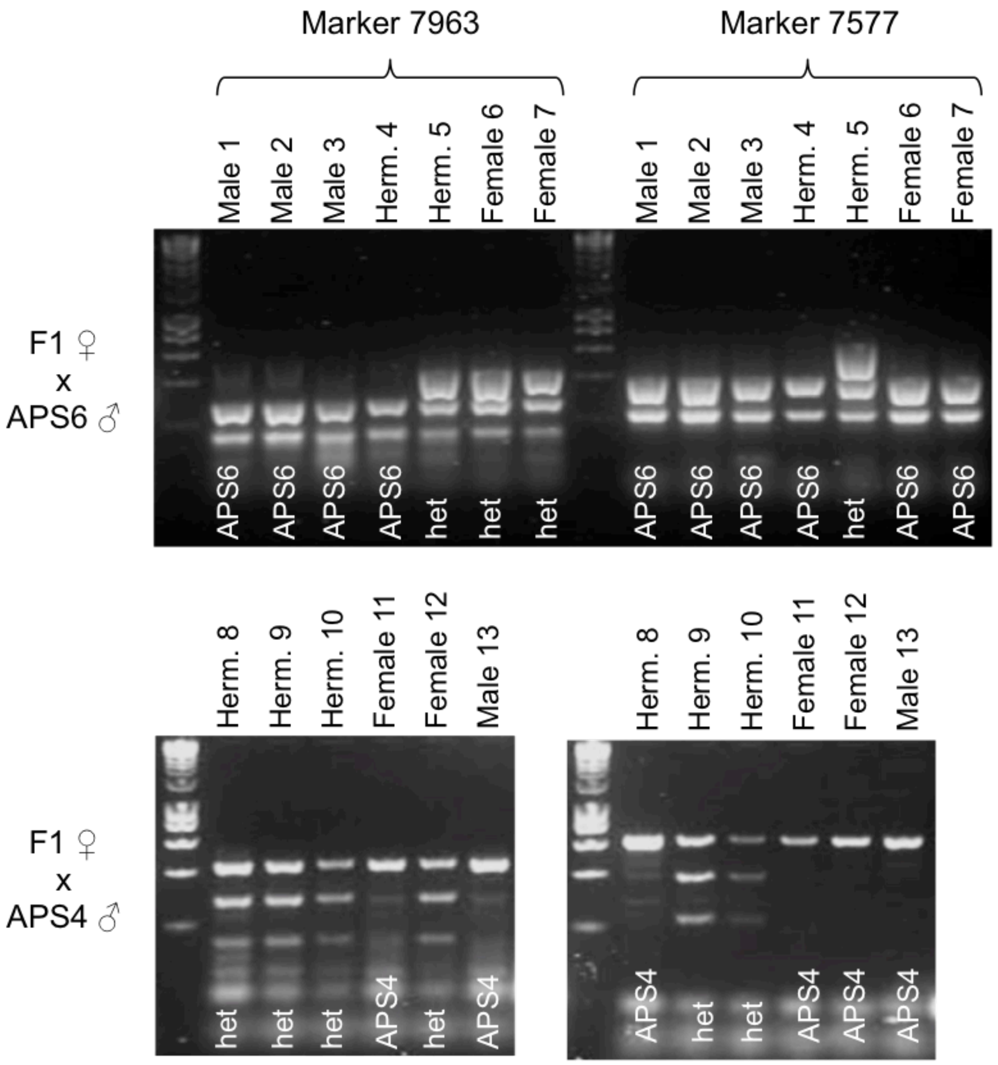

Figure S1, related to Figure 3B and C. Example of X chromosome genotyping of F2s generated from crosses between F1 hybrid $X_{APS4}X_{APS6}$ females and either APS6 (upper panel) or APS4 (lower panel) males.

From this genotyping, we can infer that some crossovers have occurred during female oogenesis as some F2 XX progeny do not display the same genotype across all the markers genotyped. The gel depicts only the two rightmost X markers (see figure 3A) as the crossovers were frequently observed between these markers (probably due to the subtelomeric position of the marker 7577). Genotypes are reported under the gel pictures. 'het' stands for heterozygous. Numbers indicate individual animals.

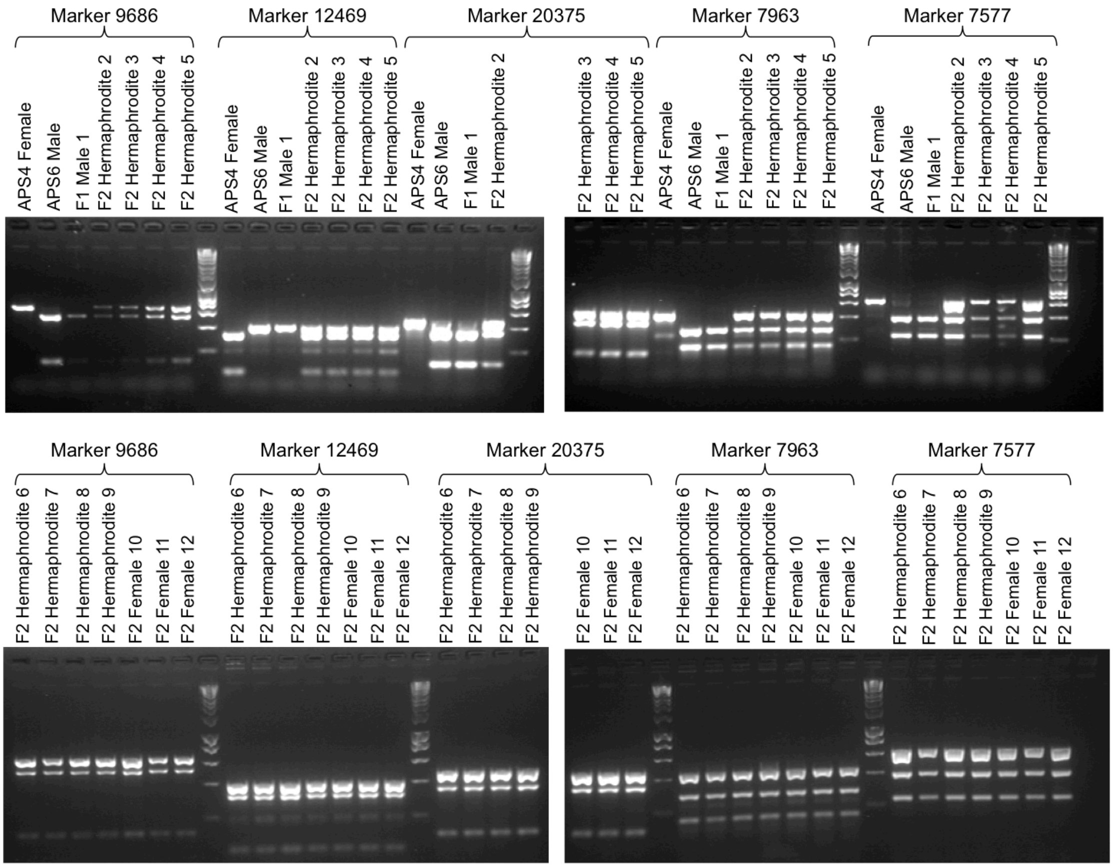

Figure S2, related to Figure 3B and D. Example of X chromosome genotyping using amplification, digestion and electrophoresis of parental individuals and F2 females and hermaphrodites produced by selfing F1 hybrid hermaphrodites.

F2 XX progeny (females and hermaphrodites) produced by hybrid F1 hermaphrodites are systematically heterozygous across the 5 X markers genotyped. Numbers indicate individual animals.

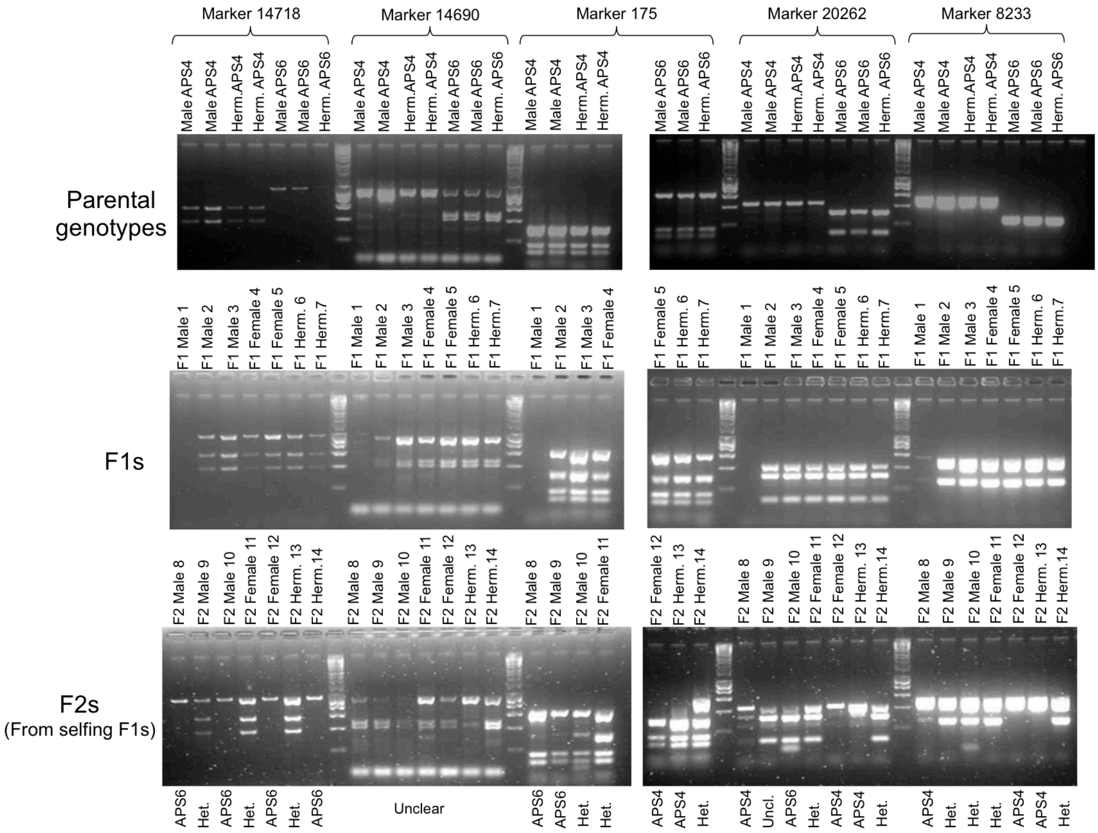

Figure S3, related to Figure 3. Example of LG4 (autosome) genotyping using amplification, digestion and electrophoresis.

F2 genotypes are reported under the gel pictures. 'Het.' and 'Uncl.' stand for heterozygous and unclear genotypes, respectively. Numbers indicate individual animals.
